# Tapered Optical Fibers Coated with Rare-Earth Complexes for Quantum Applications

**DOI:** 10.1021/acsphotonics.2c00330

**Published:** 2022-07-28

**Authors:** Ori Ezrah Mor, Tal Ohana, Adrien Borne, Yael Diskin-Posner, Maor Asher, Omer Yaffe, Abraham Shanzer, Barak Dayan

**Affiliations:** † Department of Chemical and Biological Physics, Weizmann Institute of Science, Rehovot 7610001, Israel; § Department of Chemical Research Support, Weizmann Institute of Science, Rehovot 7610001, Israel; ∥ Department of Molecular Chemistry and Material Science, Weizmann Institute of Science, Rehovot 7610001, Israel; ⊥ Department of Chemical and Biological Physics, Weizmann Institute of Science, Rehovot 7610001, Israel

**Keywords:** tapered optical fibers, RE ions, RE complexes, fluorescence spectroscopy, metallacrowns

## Abstract

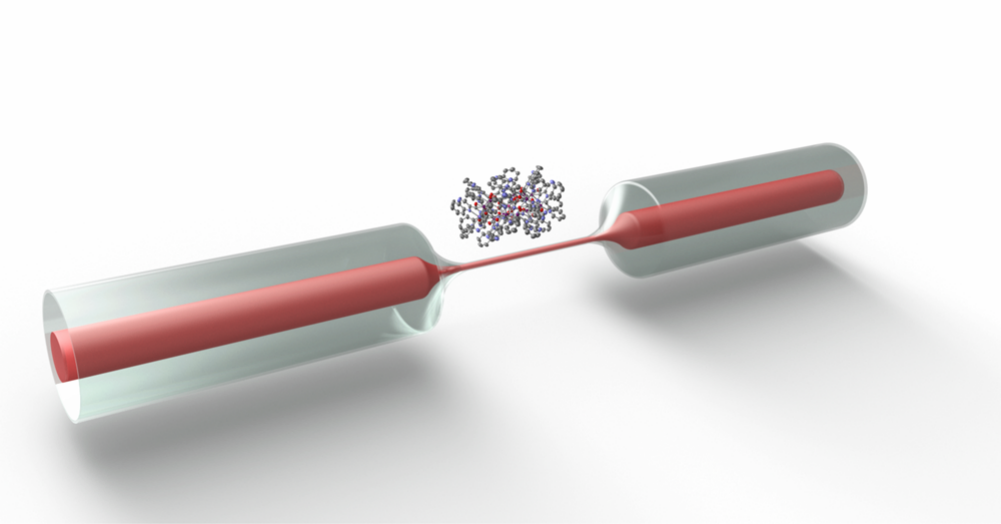

Crystals and fibers doped with rare-earth (RE) ions provide the basis for most of today’s solid-state optical systems, from lasers and telecom devices to emerging potential quantum applications such as quantum memories and optical to microwave conversion. The two platforms, doped crystals and doped fibers, seem mutually exclusive, each having its own strengths and limitations, the former providing high homogeneity and coherence and the latter offering the advantages of robust optical waveguides. Here we present a hybrid platform that does not rely on doping but rather on coating the waveguide—a tapered silica optical fiber—with a monolayer of complexes, each containing a single RE ion. The complexes offer an identical, tailored environment to each ion, thus minimizing inhomogeneity and allowing tuning of their properties to the desired application. Specifically, we use highly luminescent Yb^3+^[Zn(II)_MC_ (QXA)] complexes, which isolate the RE ion from the environment and suppress nonradiative decay channels. We demonstrate that the beneficial optical transitions of the Yb^3+^ are retained after deposition on the tapered fiber and observe an excited-state lifetime of over 0.9 ms, on par with state-of-the-art Yb-doped inorganic crystals.

## Introduction

Mapping quantum states onto the hyperfine states of rare-earth (RE) ions is one of the promising platforms for quantum technologies in general and for interaction with photonic qubits in particular. The partially filled 4f shell of all the RE^+3^ ions is shielded from the environment by the outer 5s and 5p shells, thereby reducing the influence of the host lattice on intrashell f–f transitions. As a result, RE ions exhibit sharp absorption and emission spectral lines at wavelengths that range from microwave to optical and UV, even when located inside a solid-state matrix or an organic complex. Accordingly, RE-doped crystals are the key building block in most solid-state lasers and amplifiers, and RE-doped fibers provide the basis for practically all fiber lasers^1−3^ (note that a small concentration of RE ions has been detected even in undoped fibers, as reported in ref (4)). Notably, the two major platforms for interfacing RE ions with light rely on doping the host material, either crystals or fibers. Each of these platforms exhibits its own physical properties and advantages. While RE-doped crystals enable low inhomogeneity and high coherence, RE-doped fibers offer the efficiency and practicality of a confined optical waveguide. Efforts to reconcile the two approaches have proved to be nontrivial. RE-doped silica fibers exhibit charge diffusion and tunneling modes that induce large inhomogeneity and reduce the coherence time, even at cryogenic temperatures.^5−7^ Fabrication of waveguides based on RE-doped crystals requires methods such as focused ion beam milling, ion diffusion, laser writing,^8−13^ and epitaxial growth of RE-doped oxide^14^ and silicon.^15,16^ The effort of coherently incorporating RE ions into a single-mode waveguide platform is perhaps mostly significant in the field of quantum information, where RE-doped crystals at cryogenic temperatures have already been harnessed to demonstrate quantum optical memories^8,9,12,17−19^ and are also the basis for a number of proposals for optical to microwave qubit conversion.^20^ One of the most advanced platforms in this direction is a nanophotonic cavity fabricated in a YVO_4_ crystal doped with Nd^3+^ or Yb^3+^ ions exhibiting long coherence time and narrow inhomogeneous broadening,^13^ which has been harnessed to demonstrate a quantum memory,^21^ a cavity-protected interface between RE and photonic qubits,^22^ a readout of a photonic qubit stored in a single ion in a single-shot measurement,^23^ and an optical interface between RE ions in a cavity and adjacent nuclear spins for quantum memory and entangled states.^24^ Additionally, Purcell enhancement was demonstrated with a ring resonator fabricated in Yb-doped silicon nitride.^25^

Recently, molecular complexes have been considered a promising host for metal ion qubits, such as transition metal spins,^26−28^ and also for RE ions.^29,30^ They offer control over the immediate environment of the ions at the single-atom level; thus the optical properties can be tuned as necessary. In particular, the approach enables a uniform environment to all ions, a desirable property for quantum applications. A key difference between RE complexes and RE-doped crystals is the presence of high-frequency vibrations in the complexes’ structure, which can quench the excited state and reduce the quantum yield, namely, introduce dissipation and reduce the probability of an optical emission.^31^ Therefore, highly luminescent RE complexes are designed to minimize the number of high-frequency vibrations (mainly X–H bonds with a frequency > 3000 cm^–1^) in the structure and to maximize the distance between them to the RE ion.

**Figure 1:**
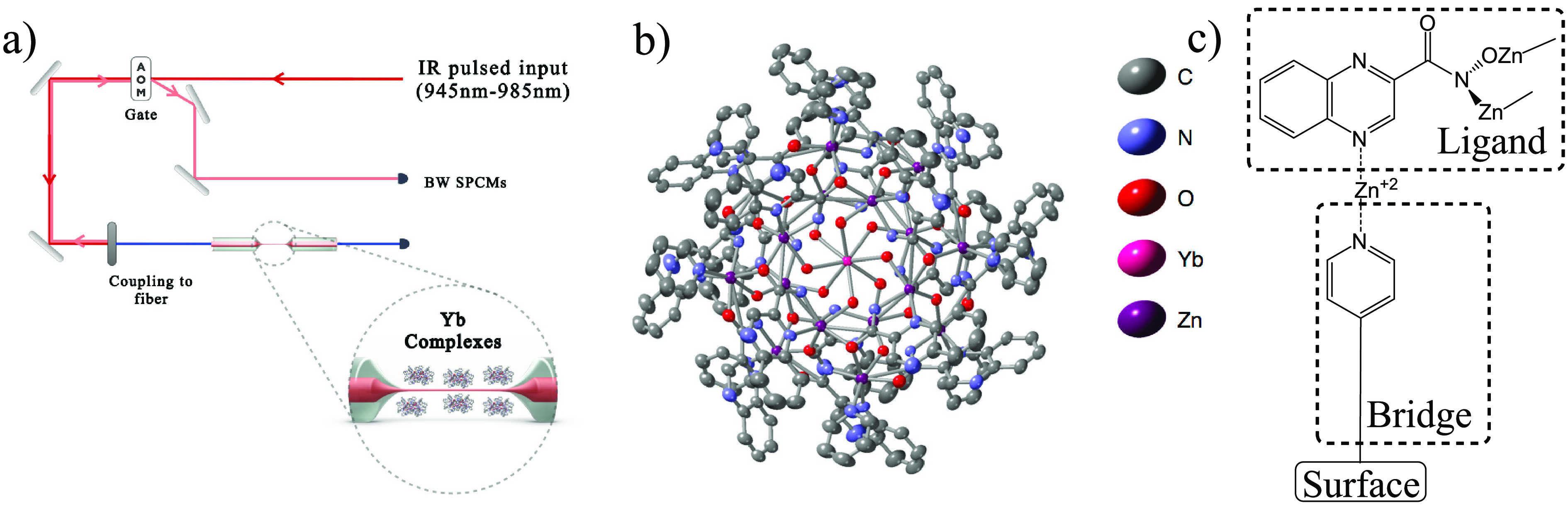
Device of this study: (a) a tapered single-mode fiber coated by a monolayer of tailored rare-earth (RE) complexes, each containing a single RE ion, is spliced to the optical setup for photoluminescence excitation (PLE) spectroscopy; ∼1 μs pulses are generated from a tunable laser (945–985 nm) and sent to the TOF (red arrow). The PL signal is collected on SPCMs in the backward (BW) direction (light red arrow) and is separated from parasitic laser reflections using a single-pass AOM serving as a gate. (b) Structure of the complex, Yb^3+^[Zn(II)_MC_(QXA)], obtained by XRD (thermal ellipsoid structure presentation with a probability of 50%; hydrogens are omitted for clarity). (c) For binding the complex to the surface, the latter is first functionalized with a pyridyl end-group, then Zn^+2^ salt is used to bridge the surface and the vacant pyridyl nitrogen of the complex (see Figure S1 in the SI for a detailed description).

Here we present a new approach for coupling RE complexes with tapered optical fibers (TOFs). TOFs allow optical interface with matter in their vicinity through the evanescent field. Such fibers have been under active research in the field of quantum applications and spectroscopy, particularly with atoms trapped near the surface^32,33^ as well as crystals of molecular dyes addressing a single dye molecule.^34^ We coat the TOF with Yb^3+^[Zn(II)_MC_ (QXA)] complexes (Figure 1a and b) that emit at the near infrared (NIR). We form a self-assembled monolayer (SAM) of our complex by functionalizing the surface of the TOF with a pyridyl end-group (Figure 1c). An important benefit of our approach is that it does not involve doping of the raw material or custom-fabrication process of the waveguide.

Since, in contrast to doped inorganic crystals, nonradiative loss is a major issue when discussing complexes, we consider the applicability of this device to quantum applications in terms of its intrinsic quantum yield. Specifically, we present optical measurements exhibiting photoluminescence (PL) decay times of over 0.95 ms, which are comparable to reported devices that are based on inorganic crystals.

## Results and Discussion

Our work includes synthesis and crystallization of a specially designed Yb complex and formation of a SAM on the surface of a TOF, resulting in record PL lifetimes in comparison to other reported Yb organic complexes. In the next subsection we discuss the synthesis and characterization, followed by the formation of the SAM, and finally we describe the luminescence properties of the bulk crystals and the functionalized TOF.

### Synthesis and Characterization of Yb^3+^[Zn(II)_MC_(QXA)]

**Figure 2:**
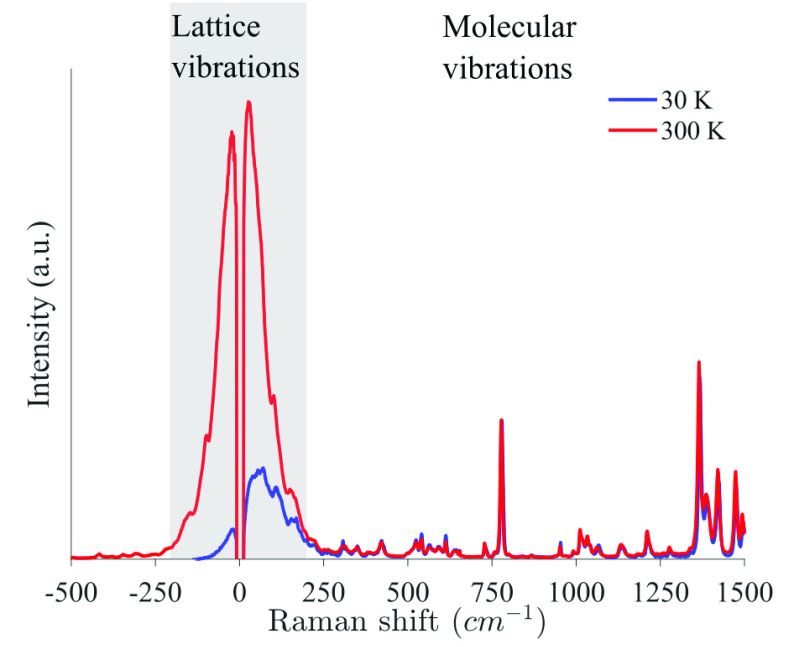
Temperature-dependent Raman spectra of the Yb^3+^[Zn(II)_MC_(QXA)] crystalline bulk, under excitation at λ_ex_ = 785 nm at room and at cryogenic temperatures (red and blue, respectively). The spectrum is divided into two regimes: the low-frequency (10–200 cm^–1^) [gray] and the high-frequency molecular vibrations (>200 cm^–1^) [white]. The spectra were normalized to the peak at 778 cm^–1^.

**Figure 3:**
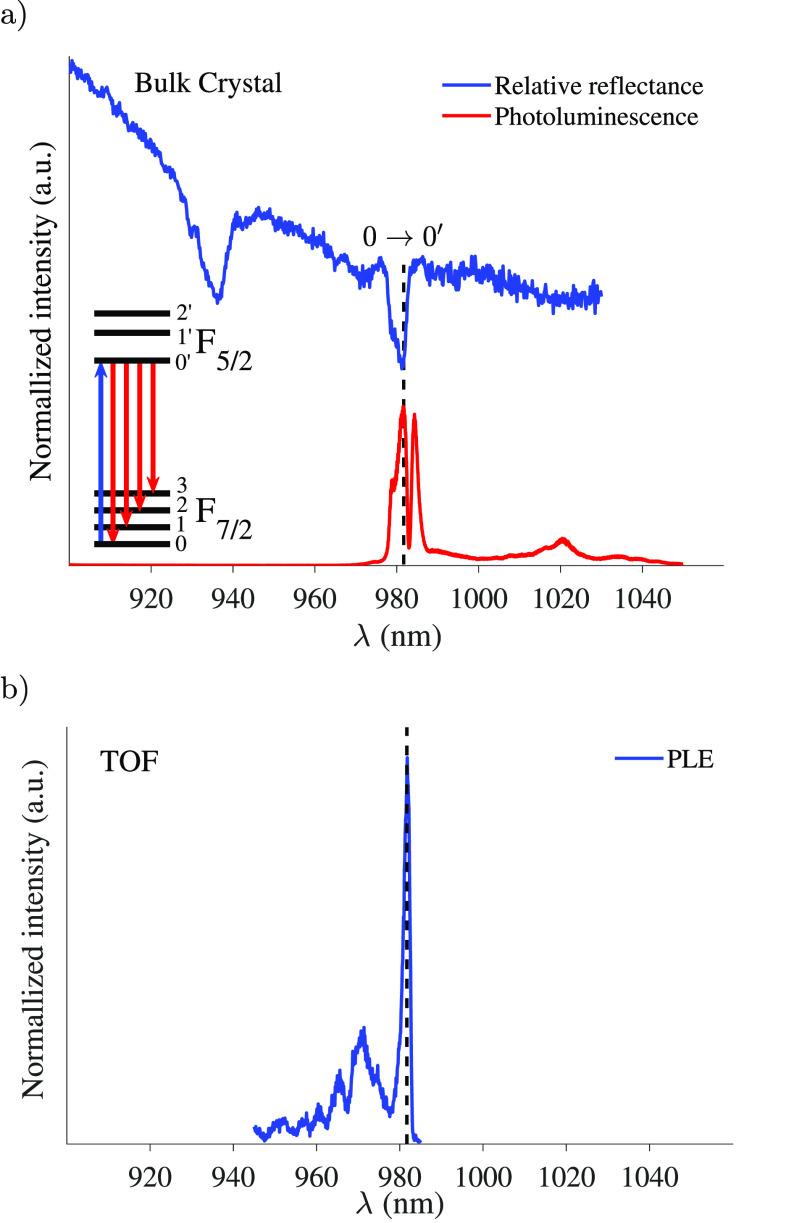
Optical spectroscopy of Yb^**+**3^[Zn(II)_MC_(QXA)] complexes at cryogenic temperatures: (a) Relative reflectance (blue) and PL (red) spectroscopy of crystalline bulk (λ_ex_ = 785 nm) at *T* ≈ 30 K and (b) PLE spectrum of a TOF coated with a monolayer of the complexes at *T* < 10 K. The PLE spectrum was taken within the laser tunability window of 945–985 nm. The main transition width is estimated from a Gaussian model with σ = 0.85 nm. Vertical line at 981.7 nm marks the mutual transition. Therefore, the Yb transitions exhibit a negligible Stokes shift and are insensitive to the deposition method on its own.

The main quenching mechanism of the luminescence originates from coupling to high-energy vibrations such as C–H, O–H, and N–H, which are common in organic ligands.^31^ Therefore, for high intrinsic quantum yield, H atoms must not be adjacent to the ion. An additional property desired for quantum information is a relatively large separation between adjacent Yb ions in order to reduce dipole–dipole interactions between them. Considering the above, we based our device on the metallacrown family of complexes, which was first synthesized by Pecoraro et al.^35^ These complexes consist of rings formed by heavier atoms—a transition metal (such as Zn^2+^), oxygen, and nitrogen—reminiscent of crown ethers. As a result, hydrogen atoms are absent from the central ion vicinity; thus nonradiative decay channels are suppressed.^36^ In order to allow binding to the surface of the TOF, we synthesized a modified version of the metallacrown complex based on the 2-quinaldic hydroxamic acid (QHA),^36^ where we used the 2-quinoxalinehydroxamic acid (QXA) ligand^37,38^ that has an additional vacant nitrogen atom, which can be harnessed for surface binding. We synthesized the ligand from its precursor 2-quinoxalinecarboxylic acid and the corresponding metallacrown complex via a modified procedure from ref (36). A detailed description of the synthesis is given in section A of the Supporting Information (SI). The complexes were crystallized using vapor diffusion of ethyl acetate into a solution of the complexes in *N*,*N*-dimethylformamide (DMF) and pyridine.^36^ X-ray diffraction (XRD) (conducted on the crystals without drying; provided in section B in the SI) indicates that the complexes form tetragonal crystals and exhibit an isostructure of their QHA analogue: two four-membered rings bound to the central RE ion with a middle eight-membered ring. The resolved structure of the complex is presented in Figure 1 b. Raman spectroscopy of the crystalline bulk (i.e., dried single crystals) (Figure 2) reveals sharp high-frequency (>250 cm^–1^) modes attributed to the organic ligand. Importantly, the low-frequency (10–200 cm^–1^) Raman spectra exhibit a broad and diffused signal at both room and cryogenic temperatures (gray). Such a broad Raman signal in the low-frequency range indicates that the crystalline bulk exhibits a relatively large level of disorder. This is important because it indicates that the relatively sharp luminescence spectrum of the bulk (shown in Figure 3) is not related to the long-range order but to the electronically isolated nature of the Yb ion.

### Formation of Yb^3+^[Zn(II)_MC_(QXA)] SAM on the TOF

As illustrated in Figure 1c, the interface between the silica and the Yb^3+^[Zn(II)_MC_(QXA)] complex is generated utilizing a bifunctional layer that is silylated on one end and possessing a pyridyl moiety on the other.^39,40^ Since the tapered optical fiber is frail, the functionalization procedure was performed at ambient conditions, to avoid mechanical damage and optical loss. The cascaded procedure involves the formation of three layers: the template layer, the zinc layer, and the complex layer. The surface is first functionalized with a silane reagent containing a chain of seven atoms and ends with a pyridyl ring (the template layer). The pyridyl ring is then connected to a Zn^+2^ salt layer, which allows the binding of the complexes via the vacant quinoxaline nitrogen atoms. The detailed procedure is provided in section C of the SI. To characterize the SAM, we first functionalized a Si substrate. After forming the SAM, we used an atomic force microscopy (AFM) tip operating in a contact mode to remove an area of 0.25 μm^2^ followed by a tapping mode imaging of the removed area (Figure S2, top, in the SI). This procedure allowed us to measure the SAM thickness. As shown in Figure S2, bottom, in the SI, a typical layer is of approximately 3 nm thickness, which is compatible with a 1.5 nm sized complex that lies on a chain of 12 atoms. It is important to note that the thickness of the SAM varied between 1.8 and 8 nm, but this is a reasonable variation for its purpose. This method is also applicable to other substrates such as alumina and can be also extended to a multilayered structure by alternately repeating the Zn salt and complex layers.

### Optical Properties of the Crystalline Bulk and Functionalized TOF

Our primary goal in this work is to demonstrate that the beneficial optical properties of RE complexes can be utilized on a functionalized TOF. Therefore, we first explore the optical properties (i.e., relative reflectance and PL; see section E in the SI for the experimental details) of the Yb^3+^[Zn(II)_MC_(QXA)] crystalline bulk (Figure 3a) and then compare them to the optical properties of the functionalized TOF (Figure 3b).

Interaction with the zero-phonon lines (ZPLs) is crucial for quantum applications and needed for a more precise assessment of the absorption and PL spectra in the crystalline bulk as well as in the monolayer. Therefore, we conducted our measurements at cryogenic temperatures. As shown in the SI (Figure S5), there is a significant broadening of the peak at *T* > 70 K.

The PLE experiment was conducted at a temperature below 10 K, as indicated by the disappearance of the transition at 984 nm. The reflectance and PL spectra were obtained using a closed chamber under a He atmosphere. From previous measurements, we estimate the temperature of the chamber at about 30 K (see the SI for details).

We focus on the main transition, which consists of an inhomogeneously broadened ensemble of Yb ions having spin degenerate pairs (a four-level system), as well as a hyperfine structure for some of the isotopes. This system is sufficient for most quantum applications. The blue trace in Figure 3a is the relative reflectance spectrum of the crystalline bulk. The bulk thickness was of a few mm, resulting in an interference pattern on the spectrum. The spectrum shows two main dips in reflectance at 981.7 nm with a shoulder at 979 nm and an additional dip at 935 nm. On the basis of the electronic configuration of Yb^3+^ (inset in Figure 3a) and a previous study on a closely related complex,^36^ we assign the transition at 981.7 nm to the 0 → 0′ transition and the other dips to transitions to higher levels in the excited states’ manifold. Additional support for the assignment of the 0 → 0′ transition comes from the PL spectrum (red trace in Figure 3a), which exhibits two main emissive transitions at 981.7 and 984.3 nm. The former transition is very close in frequency to the main transition that is observed in the relative reflectance spectrum (marked by a dashed line), indicating that the emission Stokes shift is very small. The 984.3 nm transition in the PL spectrum may represent the 0′ → 1 transition. Weaker peaks are observed at ∼1020 nm, which correspond to transitions to the higher levels in the ground-state manifold 0′ → 2 and 0′ → 3. Next, we characterized the optical transitions in the functionalized TOF. To do so, the TOF was connected to a pulsed photoluminescence excitation (PLE) spectroscopy optical setup by fiber fusion (splicing); see Figure 1 a. The integrated PL signal was collected by single-photon-counting modules (SPCMs) in the backward direction (with respect to the pump; marked by light red arrows), and any parasitic reflection of the excitation pulse was gated using an acousto-optic modulator (AOM) in a single-pass configuration (which means the first ∼1 μs of the backward signal was blocked; see section E in the SI for more details).

Given the dimensions of the TOF (a ∼5 mm long waist with a diameter of ∼500 nm), the estimated number of emitters is less than ∼10^10^ (see section D in the SI for more details). Note that the geometry and frailty of the TOF prevented us from performing the same measurements we performed on the bulk crystal or on the silicon chip monitor.

The blue trace in Figure 3b presents the PLE spectra of the functionalized TOF at cryogenic temperatures obtained by the immersion in a liquid He dewar. The spectrum shows a main peak at 981.7 nm. A broader feature appears between 960 and 977 nm. The most important finding in the context of this study is that the peak at 981.7 nm, which is assigned to the 0 → 0′ and marked by a dashed line, is sharp and dominant. This demonstrated that the absorption spectrum of the Yb^3+^[Zn(II)_MC_(QXA)] complex was unaffected by the deposition on the TOF. Moreover, it is narrower than the corresponding transition in the crystalline bulk. We therefore conclude that the two directions (absorption and emission) of the main transition are effectively unchanged for the two deposition methods. It also possesses a negligible Stokes shift between absorption and emission, which implies a relatively pure transition (i.e., uncoupled to vibrations). The precise assignment of all peaks requires further study of the optical behavior of these complexes.

Our aim is to reach a comparable lifetime to Yb inorganic doped crystals, which is on the order of 1 ms (depending on the host crystal).^41,42^ At ambient conditions, the time-resolved PL of the coated fiber exhibits a lifetime of 4 μs, much shorter than the lifetime of Yb-doped inorganic crystals. On the basis of the work of Pecoraro et al.,^36^ we hypothesized that the origin of the shorter lifetime in this system is the adsorbed solvent molecules on the layer. These solvents can be removed by placing the monolayer under vacuum. Since the complexes consist of labile N–O bonds, they are susceptible to thermal decomposition. We therefore avoided bake out at elevated temperatures.

**Figure 4:**
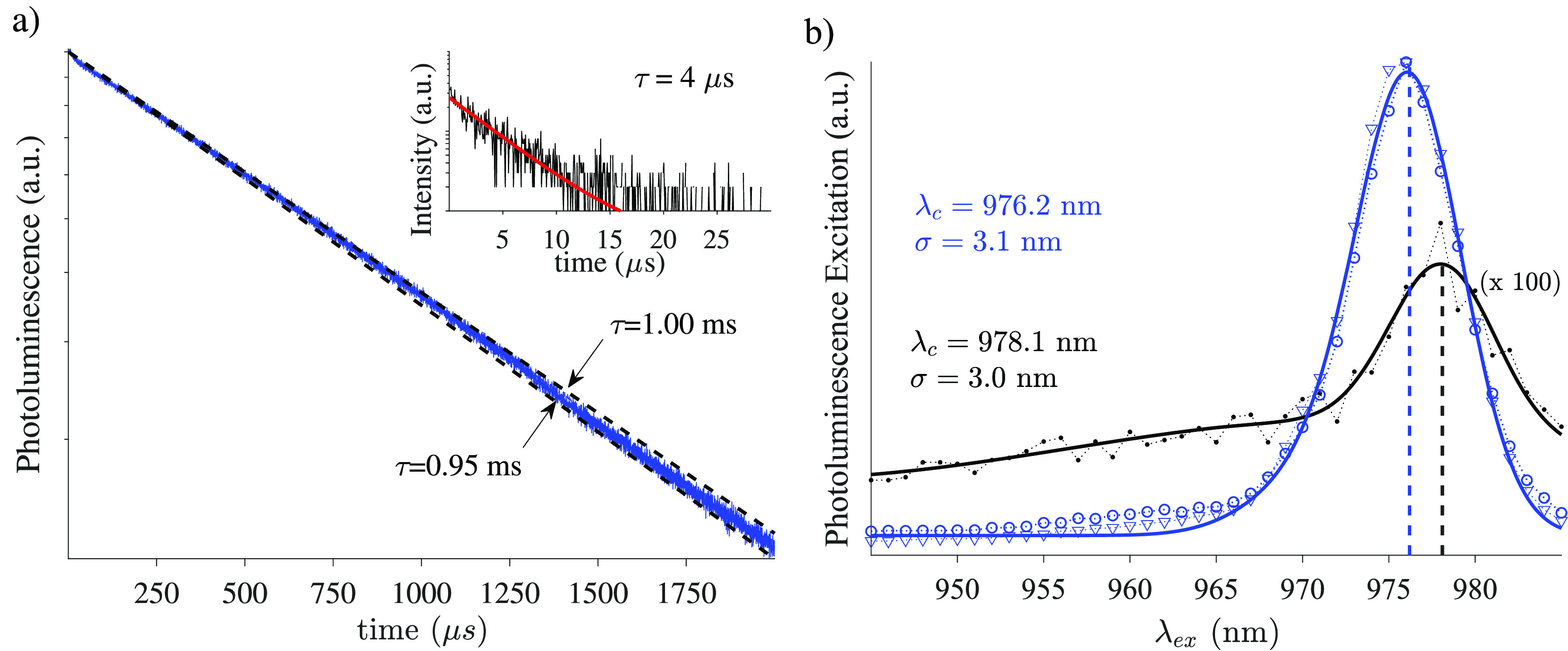
(a) Decay of the PL signal upon excitation on resonance of a TOF functionalized with a monolayer of Yb^3+^[Zn(II)_MC_(QXA)] complexes. While at ambient pressure (inset) the monolayer exhibited a time constant of a few μs, after a few days in high vacuum, the time constant exceeds 0.95 ms (blue). (b) PL excitation spectra measurements for different samples of fibers coated with a monolayer of Yb^3+^[Zn(II)_MC_(QXA)]. Solid lines represent fits. One of the samples was measured at atmospheric pressure (black dots). Both samples were measured after over a week under vacuum (blue circles and triangles). The black curve was multiplied by 100 for better visibility. The fits (see the SI for details) show a main resonance at 978 and 976 nm (at atmospheric pressure and under vacuum, respectively). The main peak at 978 nm corresponds to the 0 → 0′ transition shifts to 976 nm under vacuum. This is attributed to the removal of ligating solvent molecules, which changes the level splitting of the Yb ion.

Indeed, by introducing the TOF into high vacuum (as low as 10^–7^ mbar), we extended the lifetime drastically. The blue traces in Figure 4 show the time-resolved PL (a) and PLE spectrum (b) of a functionalized TOF after a few days (typically a week) under high vacuum (10^–7^ mbar). We observe a dramatic increase in lifetime by over 2 orders of magnitude: from approximately 4 μs at ambient conditions to over 0.95 ms. Accordingly, the overall fluorescence increased by nearly 2 orders of magnitude. These changes were accompanied by a shift of the main transition from 978 to 976 nm, which results from the modification in the Yb environment. It is interesting to compare these results to those obtained using commercial Yb-doped fibers. As shown in Figure S4 of the SI, the doped fiber exhibited a somewhat shorter lifetime (∼0.84 ms) and a wider PLE spectrum.

This work presents low-temperature spectra with an inhomogeneous line width estimated at ∼530 GHz (σ = 0.85 nm fitted with a Gaussian function). This value is 2–3 orders of magnitude larger than the line widths obtained with a low concentration of Yb-doped crystals, commonly used for quantum applications (e.g., 2.2 GHz in YSO,^42^ 0.275 GHz in YVO_4_,^43^ and 3.6 GHz in YAG^44^). On the other hand, there are Yb complexes such as Yb(dpa)_3_^45^ that show comparable inhomogeneous line widths in their crystal form at 15 K. We believe the wider line width in our case is mainly a result of near-field interactions due to the high density of Yb ions in the monolayer (distance of approximately 1.5 nm between adjacent ions, according to the crystal structure). In terms of lifetime, inorganic doped crystals exhibit lifetimes of 0.267 ms,^43^ 0.87 ms,^42^ and 1.0 ms,^44^ and a few Yb doped crystals (such as YLF^46^ and CaF_2_^47^) exceed the 1 ms lifetime. In organic Yb-based NIR fluorophores, the longest reported lifetime is 0.7 ms (via sensitization).^48^ In comparison, our device exhibits a 0.95 ms lifetime, which is well within the lifetime range of doped crystals. Moreover, note that the fluorescence emitted to the fiber is expected to be enhanced due to the fiber’s cooperativity (estimated by 0.1–0.2^49,50^). Accordingly, the obtained value of ∼0.95 ms corresponds to an even slightly longer decay time in free space, which indicates that our device exhibits efficient isolation of the Yb^3+^ ions from nonradiative decay channels.

Interestingly, the monolayer structure also provides a route to control the distance between adjacent RE ions, as well as from the glass surface, thus reducing the effect of decoherence sources originating from the glass. In order to reduce the RE ion density in the layer, one could modify the complex layer, for example, by synthesizing a bulkier complex (such as a similar metallacrown with side groups larger than quinoxaline) or dilute the complexes by a mixture of complexes of Yb and optically inert RE ions such as La, Lu, and potentially Gd (whose lowest energy transition is in the UV).

This platform is generally applicable to all RE ions and can be applied on any photonic device made of oxygenated substrates such as Si, glass, or Al_2_O_3_ and can be applied to other highly fluorescent RE complexes that can form a SAM; it enables enhanced coherent coupling to RE ions utilizing the evanescent field of waveguides and whispering-gallery-mode resonators, potentially opening the path toward a large number of optical and quantum-optical applications. Further characterization of the coherence properties of this device (in particular its homogeneous line width) is required in order to substantiate its suitability for quantum applications.

## Conclusions

In conclusion, we present a new interface between RE ions and TOFs, based on organic complexes with low nonradiative loss, i.e., high intrinsic quantum yield. We observed similar spectra of the encapsulated Yb^3+^ intra 4f-transitions in different systems: a crystalline bulk and a monolayer. We further observed optical coupling of a monolayer of complexes on a TOF placed in high vacuum, with τ > 0.95 ms, which confirms the efficient suppression of nonradiative decay channels, comparable to Yb-doped inorganic crystals.

## Supporting Information Available

The synthetic procedure for the ligand and complex, as well as the surface functionalization procedure, additional optical measurements, and details of the tapered optical fibers (PDF)

## Notes

The X-ray crystallographic coordinates for the structure reported in this study have been deposited at the Cambridge Crystallographic Data Centre (CCDC), under deposition number 2102073. The data can be obtained free of charge from The Cambridge Crystallographic Data Centre via https://www.ccdc.cam.ac.uk/data_request/cif.
