# Supplementary material for: Tapered Optical Fibers Coated with Rare-Earth Complexes for Quantum Applications
Source: ACS Photonics. 2022 Jul 28;9(8):2676–82. doi: 10.1021/acsphotonics.2c00330 (PMC9390790; doi:10.1021/acsphotonics.2c00330)
Supplement: Supplementary file 1 — ph2c00330_si_001.pdf [file ph2c00330_si_001.pdf]

Supporting information for Tapered optical fibers coated with Rare-Earth complexes for quantum applications

Ori Ezrah Mor,[1] Tal Ohana,[1] Adrien Borne,[1,2] Yael Diskin-Posner,[3]
Maor Asher,[1] Omer Yaffe,[1] Abraham Shanzer,[4] and Barak Dayan*[1]

[1] *Department of Chemical and Biological Physics, Weizmann Institute of Science, Rehovot 7610001, Israel*

[2] *Current affiliation: Université de Paris, Laboratoire Matériaux et Phénomènes Quantiques, CNRS UMR7162, F-75013 Paris, France*

[3] *Department of Chemical Research Support, Weizmann Institute of Sceince, Rehovot 7610001, Israel*

[4] *Department of Molecular Chemistry and Material Science, Weizmann Institute of Science, Rehovot 7610001, Israel*

A. Synthesis

1. *2-Quinoxaline Hydroxamic acid*

Potassium hydroxide was purchased from, Macron fine chemicals.
Hydroxylamine hydrochloride was purchased from Fluka.
Anhydrous dichloromethane (DCM), Ethyl chloroformate, and 4-methyl morpholine were purchased from Sigma.
2-Quinoxaline carboxylic acid was purchased from Apollo Scientific.
NMR spectroscopy was conducted on Bruker UltraShield AVANCE III-300 MHz spectrometer, and referenced with respect to TMS (In proton NMR) and solvent (In ^{13}C NMR).
In a vial, KOH, 308 mg (5.49 mmol), were dissolved in 5 ml MeOH. In a separate vial, $NH_2OH \cdot HCl$, 381 mg (5.48 mmol , 1.5 eq) were dissolved in 5 ml MeOH. Both solutions were then cooled using an ice bath, combined, and stirred for $\sim$ 25 min at 0^0C. They were then filtered and the filtrate was set aside in an ice bath.
Meanwhile, in an oven dried apparatus, under Ar, 2-Quinoxaline carboxylic acid, 637 mg (3.66 mmol) were dissolved in 50 ml anhydrous DCM resulting in a blurred suspension with a very light pink-brownish color. Then, 4-methyl morpholine, 0.44 ml (4.0 mmol, 1.1 eq) were added and the suspension immediately clarified with a dark brown color. The solution was stirred until all solids disappeared. Afterwards, the solution was cooled using an ice bath and Ethylchloroformate 0.45 ml (4.7 mmol, 1.3 eq) were added. The solution was stirred for about 35 min. Then, the NH_2OH solution was added and the combined solution was stirred at 0^0 C for 45 min. The solution was then allowed to reach room temperature and was stirred for additional $\sim$ 3h. Then, the solution was filtered and concentrated at the pump. It was then added to 50 ml of DI water (Merck, HPLC grade) and overnight, solids were formed. They were filtered at the pump and dried in vacuum.
Yield: 253 mg (36%)
HNMR
(300 MHz, CD_3SOCD_3) δ, 11.85 (s, 1H), 9.45 (d, 1H), 8.23 (m, 2H) , 8.04 (m, 2H) (Fig. S6).
CNMR
CD_3SOCD_3 DMSO-d6 75 MHz, δ 160.6, 144.7, 143.6, 142.8, 139.8, 131.8, 131.2, 129.4, 129.1 (Fig. S7).
ESI-MS, (m/z)=190.0617 (M+H), $C_9H_7N_3O_2$,calc. Mass (189.0538)

2. *Yb^{+3}[Zn(II)$_{MC}$(QXA)] triflate*

N,N-dimethylformamide (DMF), triethylamine, Zinc(II) triflate and Yb(III) triflate were purchased from Sigma Aldrich.
2-Quinoxaline hydroxamic acid, 70 mg (0.37 mmol), Zinc triflate, 135 mg (0.37 mmol), and Yb triflate, 31 mg (0.05 mmol) were dissolved in 5 ml DMF. The solution was yellow. Then, trimethylamine, 0.1 ml were added and the solution suddenly became dark red. It was stirred overnight at RT. The following day, 5 ml pyridine were added and the solution was left to crystallize by vapor diffusion of ethyl acetate.
The crystals were filtered at the pump, and then placed in vacuum overnight. The yield is 4 mg.
ESI-MS (Z=3), m/z=1404.46 .

B. Crystal structure determination

$C_{184}H_{120}N_{56}O_{32}YbZn_{16}+2CF_3O_3S$ and solvent, orange prism, 0.166 x 0.146 x 0.103 mm^3, tetragonal $P4/ncc$, $a = b = 29.5104(3)\AA$, $c = 27.3204(4)\AA$, $\alpha = \beta = \gamma = 90^o$, from 521923 reflections and 26° data, $T = 100(2)$ K, $V = 23792.3(6)\AA^3$, $Z = 4$, $Fw = 5161.46$, $Dc = 1.441$ $Mg\cdot^{-3}$; $\mu = 2.065mm^{-1}$.

Data collection and processing Rigaku Synergy-S dual source diffractometer equipped with microfocus and PILATUS-3R 300K detector. MoKα (λ = 0.7103$\AA$), $-36 \leq h \leq 36$, $-36 \leq k \leq 36$, $-34 \leq l \leq 34$, frame scan width = 0.5^o, scan speed 1.0^o per 360.68 sec, 521923

reflections collected, 12116 independent reflections (R-int=0.0815). The data were processed with CrysAlis^{PRO}.

Solution and refinement
Structure was solved with SHELXT program. Full matrix least-squares refinement based on F^2 with SHELXL on 715 parameters with 70 restraints gave final $R_1 = 0.0470$ (based on F^2), wR_2 = 0.1235 for data with $I \geq 2\sigma(I)$, and $R_1 = 0.0663$ $wR_2 = 0.1477$ for all data on 12116 reflections, goodness-of-fit on $F^2 = 1.079$ largest electron density peak 2.842 $e \cdot \AA^{-3}$. Largest hole - 0.786$e \cdot \AA^{-3}$.

C. Glass functionalization

(4-pyridylethyl)3-thiopropyl trimethoxysilane was purchased from Gelest Chemical Company.
Isopropanol and methanol (HPLC grade) were purchased from Sigma-Aldrich.
DI water (HPLC grade) was purchased from Merck.
Toluene, Sulfuric acid and hydrogen peroxide (30 %) were purchased from BioLab Inc.

Step 1

Substrates were cleaned using Piranaha solution (sulfuric acid: hydrogen peroxide solution 30% - 3:7) for 30 min **(Pirnaha is a dangerous solution)**. They were then rinsed with DI water 8 times and dried in an oven.

A solution of Toluene (10 ml), and 30 µL (4-pyridyl ethyl) 3-thiopropyl trimethoxy silane was prepared. Substrates and TOF were rinsed for approximately 5 min and were then washed with toluene 8 times in 10 ml portions.

Step 2

Zinc triflate (10 mg) were dissolved in 10 ml 2-propanol.

The substrates and TOF were rinsed for approximately 5 min in the solution and were then washed with isopropanol 5 times in 10 ml portions.

Step 3

A stock solution of Yb^{+3}[Zn(II)$_{MC}$(QXA)] triflate (∼ 1 mg) in 10 ml isopropanol was prepared.

For every experiment, 30 µL from the stock solution were added to 10 ml isopropanol.

The substrates and TOF were rinsed for approximately 5 min and were then washed with isopropanol 5 times in 10 ml portions and then additional portion of 10 ml Methanol.

Note: Attempts to perform the last step from a pure methanolic solution resulted in the formation of a very thin layer – less than 1 nm which suggests that the methanol dissolved the template layer.

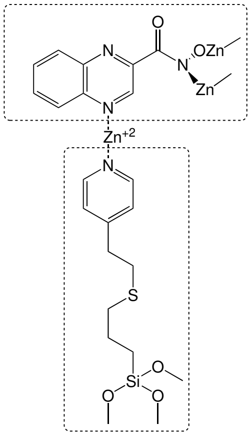

FIG. S1. **Detailed schematic description of binding to the surface**: The surface is functionalized with the silane reagent having a pyridyl end-group. Zn^{+2} ions bridge the pyridyl group with the vacant quinoxalyl nitrogen atoms of the complexes.

D. Fabrication of tapered optical fibers (TOFs)

The TOFs were pulled from a 780 nm single mode fiber (Nufern 780-HP). They were pulled over a Hydrogen flame (in air) at a typical constant rate of 0.02 mm per sec (0.01 mm per sec to each direction). The total elongation was approximately 24 mm, with a waist diameter estimated at 500 nm. The flame hot-zone (which is also the waist size) is about 5 mm, as verified in SEM measurements. The number of emitters on the waist, assuming a homogeneous monolayer is estimated at 10^{10} based on the 1.5 nm size of a single complex (from XRD measurement). The indication for the formation of the monolayer was obtained by characterizing a monitor Si chip which underwent the same procedure as the TOF (which is too frail to allow direct chracterization of the monolayer).

E. Optical Setup and spectroscopic methods

1. Photoluminescence excitation (PLE) spectrum on a tapered optical fiber (TOF)

The Yb^{+3} ions were directly excited into the 4f-4f transitions by optical pulses created from an external cavity tunable laser diode (NewFocus Velocity TLB-6700), in the spectral range of 945-985 nm, chopped using an Acousto-Optic Modulator (AOM) in a double-pass configuration. The gate consists of an additional AOM in a single-pass configuration. The spectra at room temperatures were obtained using pulses of typically 1 μs. The laser was scanned in a 1 nm resolution in the range of 945-985 nm. The PL signal has been collected in bins of 51.2 ns which were further binned to 1 μs. The spectra

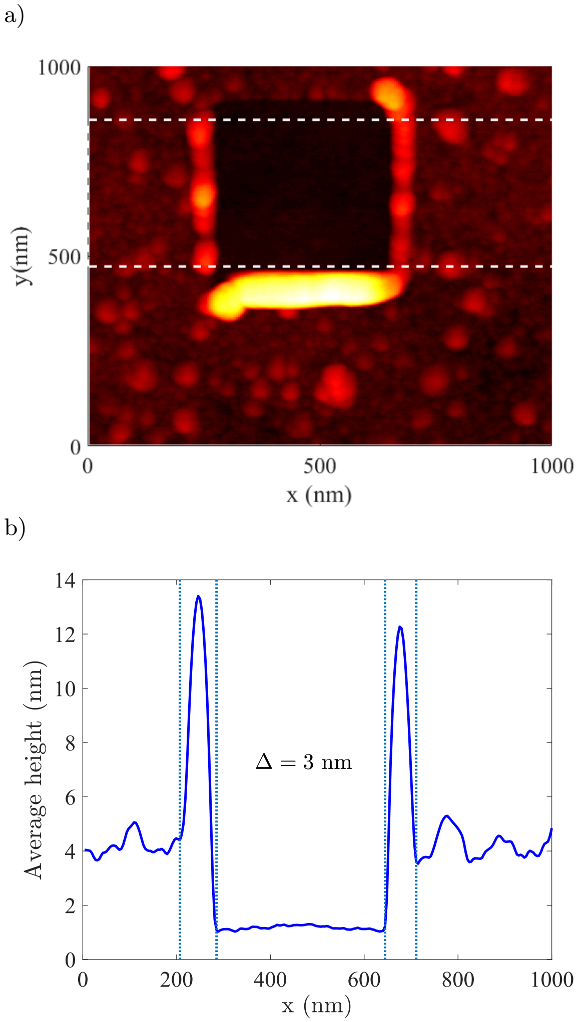

FIG. S2. (a) AFM image of a typical monolayer grown on a Si chip, and (b) the averaged cross section (calculated from the dashed white frame). The sample was first scanned in a contact mode to remove the layer, and was then scanned on a bigger area using the tapping mode. We observe average thickness of 3 nm between the hole and its surroundings.

were fitted with the following bi-Gaussian model:

$$f(\lambda) = a + b_1 \cdot exp(-(\lambda - \lambda_{c1})^2/(2s_1^2)) + b_2 \cdot exp(-(\lambda - \lambda_{c2})^2/(2s_2^2)) \quad (1)$$

The time-resolved PL was fitted with either a single or bi-exponential decay with a DC component model:

$$f(t) = a + b \cdot exp(-t/\tau_1) + c \cdot exp(-t/\tau_2) \quad (2)$$

Time-resolved PL using different pulse duration: Figure S3 depicts additional time-resolved PL in high vacuum with varying excitation pulses. As seen, the $\sim$ 0.9 ms decay is obtained in short (0.5 μs red curve) square excitation pulse, as well as in very long square excitation pulses (2 and 20 ms-blue and green curves respectively). We therefore conclude that it is indeed an inherent sample property rather than influenced by the experimental procedure.

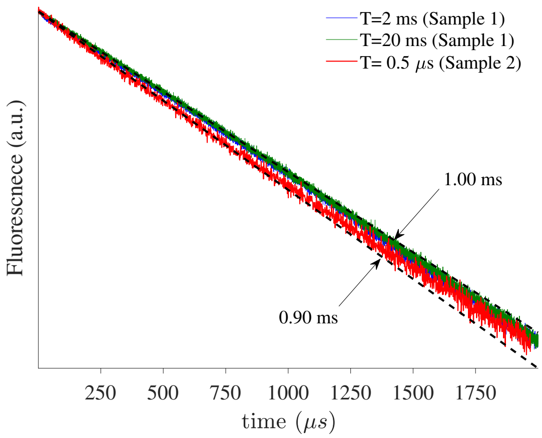

FIG. S3. Time-resolved PL with varying excitation pulse width. The dashed black lines represent exponential decay with time-constant as specified. Despite 3 orders of magnitude difference in the excitation pulse duration, the decay time constant barely changes (blue, green, and red). Red, an additional sample that underwent the same functionalization procedure.

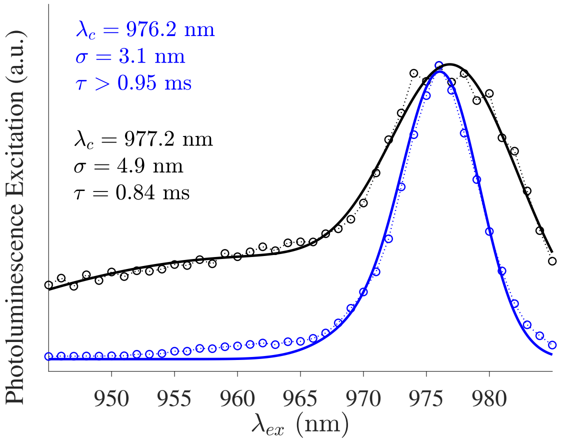

FIG. S4. PLE spectroscopy of commercial Yb doped single-mode fiber (black), and functionalized TOF in high vacuum (blue). The curves were fitted with Gaussian models (main resonance and width provided). The doped fiber exhibits somewhat larger width of the main resonance with a pronounced sideband from 945 to 970 nm (black). In comparison, the TOF exhibits less inhomogeneity and weak sideband signal. We therefore conclude that the complexes shield the Yb ions from the glass to some extent.

In fig. S4 we show the comparison between our coated TOF in vacuum, and a 2 cm segment of a commercial Yb doped single mode fiber (Thorlabs YB1200-4/125) at ambient conditions. We measured a lifetime of

$\sim$ 0.84 ms and the PLE spectrum exhibits a slightly larger inhomogeneous width in comparison to the coated TOF in vacuum.

2. Raman, PL, and reflectance spectroscopy

Raman and PL spectroscopy were obtained by excitation at $\lambda_{ex} = 785$ nm using Toptica diode laser on a home-built optical setup. We used 1 m long Horiba FHR-1000 dispersive spectrometer equipped with 600 mm^{-1} grating, and a Si CCD detector (Horiba Synapse). The dried crystals (i.e. crystalline bulk) were inserted into a sealed cell in a He atmosphere and were measured free-standing. The cell temperature was controlled using a Janis cryostat ST-500 and a temperature controller by Lakeshore model 335. The PL spectrum was corrected for the detector's response.
For the reflectance spectroscopy, the optical setup was changed by replacing the light source to a tungsten lamp (NewPort, Oriel Instruments) and the addition of a long-pass filter at 780 nm. The spectrum was referenced by the SiO_2 window of the chamber. The PL experiment was simultaneously conducted with the Raman spectroscopy of the crystal. The temperature is estimated to be $\sim$ 30 K from the level of the anti-stokes of the diffused low frequency signal. The reflectance experiment was conducted using the same cryostat, which was held at $T < 10$ K.
In figure S5 we present PL spectroscopy of the crystalline bulk of Yb^{+3}[Zn(II)$_{MC}$(QXA)] at different temperatures.

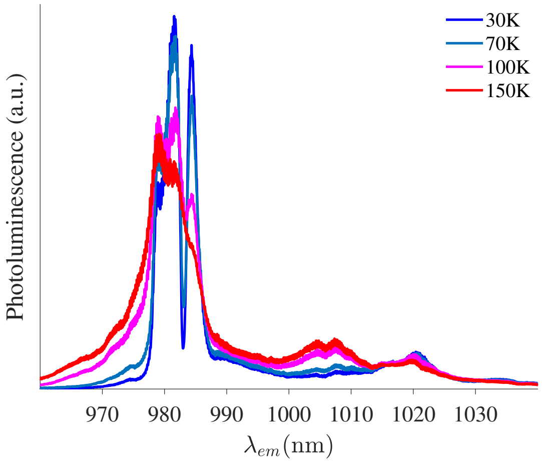

FIG. S5. PL spectroscopy of the crystalline bulk of Yb^{+3}[Zn(II)$_{MC}$(QXA)] at different temperatures. A significant peak broadening occurs at $T > 70$ K.

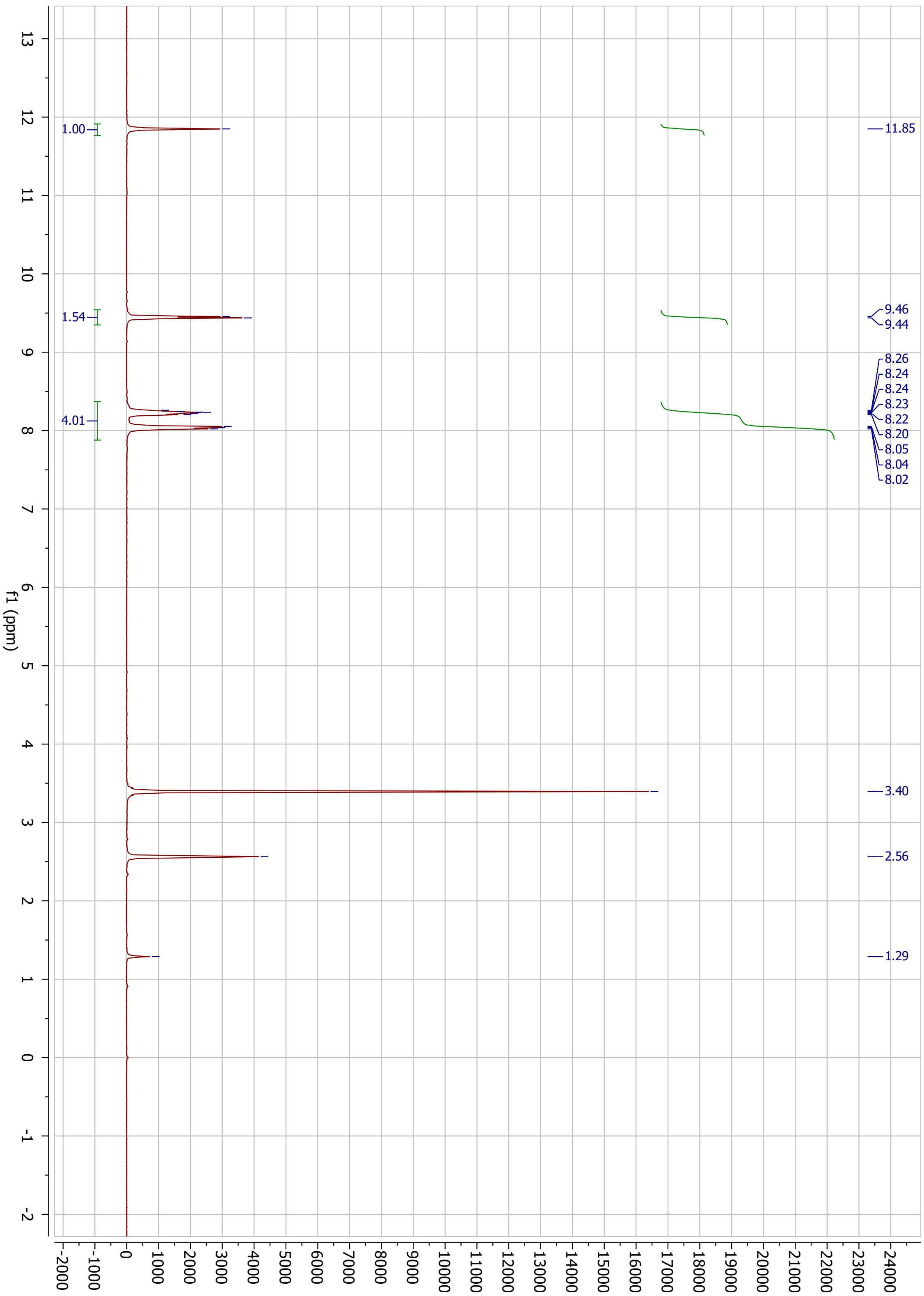

FIG. S6. HNMR spectrum of 2-Quinoxaline Hydroxamic acid

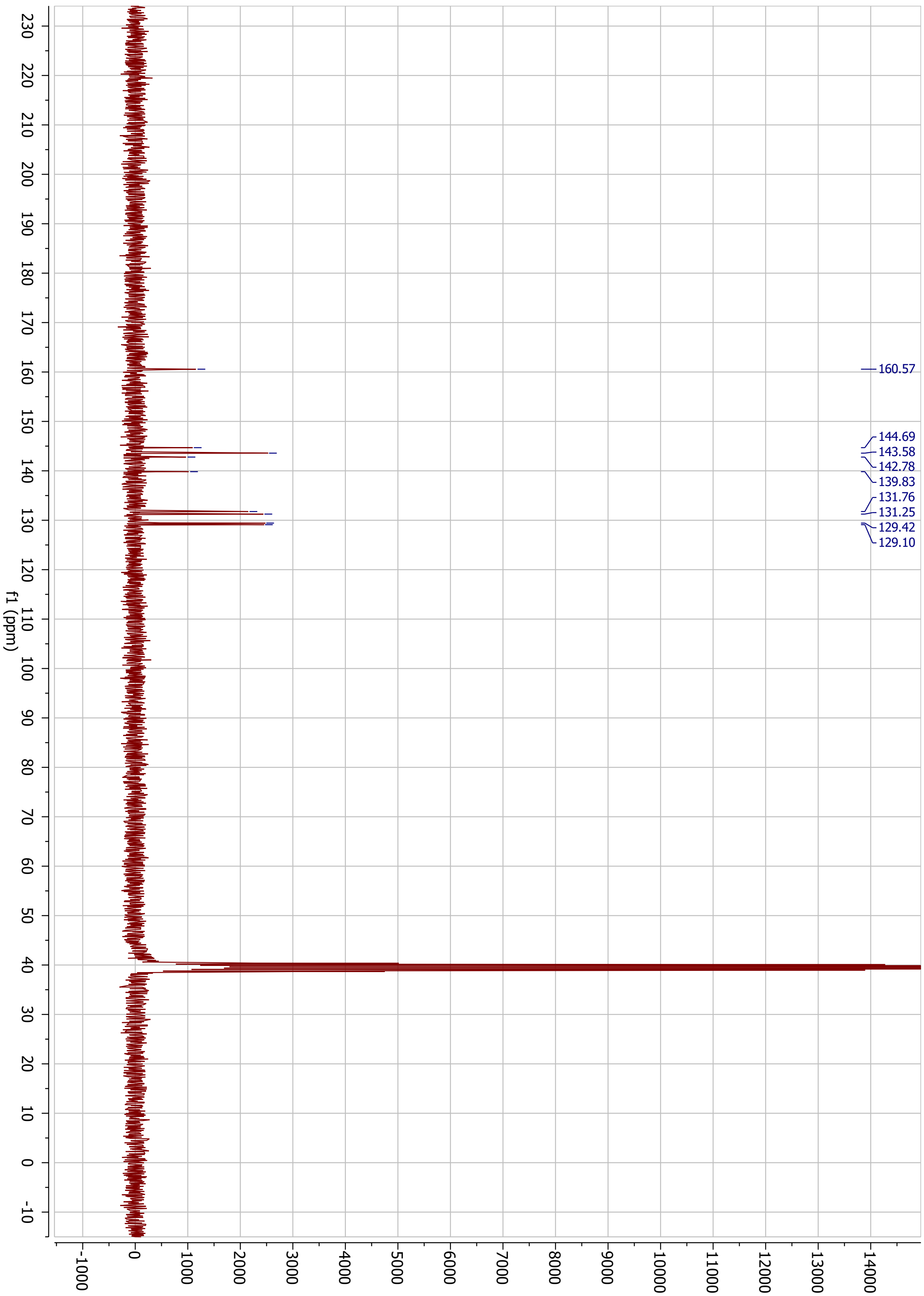

FIG. S7. CNMR spectrum of 2-Quinoxaline Hydroxamic acid
